# Analysis of the *Salmonella* regulatory network suggests involvement of SsrB and H-NS in σ^E^-regulated SPI-2 gene expression

**DOI:** 10.3389/fmicb.2015.00027

**Published:** 2015-02-10

**Authors:** Jie Li, Christopher C. Overall, Ernesto S. Nakayasu, Afshan S. Kidwai, Marcus B. Jones, Rudd C. Johnson, Nhu T. Nguyen, Jason E. McDermott, Charles Ansong, Fred Heffron, Eric D. Cambronne, Joshua N. Adkins

**Affiliations:** ^1^Department of Molecular Microbiology and Immunology, Oregon Health and Science UniversityPortland, OR, USA; ^2^Biological Sciences Division, Pacific Northwest National LaboratoryRichland, WA, USA; ^3^Department of Infectious Diseases, J. Craig Venter InstituteRockville, MD, USA

**Keywords:** *Salmonella*, RpoE, microarray, SPI-2, H-NS, regulation, ChIP-seq

## Abstract

The extracytoplasmic functioning sigma factor σ^E^ is known to play an essential role for *Salmonella enterica* serovar Typhimurium to survive and proliferate in macrophages and mice. However, its regulatory network is not well-characterized, especially during infection. Here we used microarray to identify genes regulated by σ^E^ in *Salmonella* grown in three conditions: a nutrient-rich condition and two others that mimic early and late intracellular infection. We found that in each condition σ^E^ regulated different sets of genes, and notably, several global regulators. When comparing nutrient-rich and infection-like conditions, large changes were observed in the expression of genes involved in *Salmonella* pathogenesis island (SPI)-1 type-three secretion system (TTSS), SPI-2 TTSS, protein synthesis, and stress responses. In total, the expression of 58% of *Salmonella* genes was affected by σ^E^ in at least one of the three conditions. An important finding is that σ^E^ up-regulates SPI-2 genes, which are essential for *Salmonella* intracellular survival, by up-regulating SPI-2 activator *ssrB* expression at the early stage of infection and down-regulating SPI-2 repressor *hns* expression at a later stage. Moreover, σ^E^ is capable of countering the silencing of H-NS, releasing the expression of SPI-2 genes. This connection between σ^E^ and SPI-2 genes, combined with the global regulatory effect of σ^E^, may account for the lethality of *rpoE*-deficient *Salmonella* in murine infection.

## Introduction

*Salmonella enterica enterica* serovar Typhimurium (strain 14028s; referred to as *Salmonella* hereafter) is an invasive enteric pathogen with remarkable adaptability to diverse environments. The host as well as the residential niche of *Salmonella* varies, requiring the pathogen to sense its location within the host and to adjust its gene expression accordingly. *Salmonella* has evolved a number of strategies to sense the environment and to modulate its production of virulence factors appropriately (Alpuche Aranda et al., 1992; Raffatellu et al., 2005; Yoon et al., 2009). The bacterial surface is the frontline of the host-pathogen interaction, making it both a major target of the host immune response and a primary location for the pathogen to activate its own defensive strategies (Rowley et al., 2006).

In Gram-negative bacteria, stresses that affect components of the cell envelope, such as periplasmic and outer-membrane proteins, illicit a variety of responses in the cell which are collectively known as extracytoplasmic stress responses (ESRs). There are at least four signal transduction systems in Gram-negative bacteria that govern the ESRs: the alternative sigma factor σ^E^ (encoded by *rpoE*), the two-component regulatory systems CpxR/CpxA and BaeS/BaeR, and the phage shock protein (Psp) system (4). Of these systems only the absence of σ^E^ causes a strong virulence defect, although there is overlap and crosstalk between the systems when reacting to different extracytoplasmic stresses (Connolly et al., 1997; Jones et al., 1997; Humphreys et al., 1999, 2004; Kenyon et al., 2002; Becker et al., 2005; Karlinsey et al., 2010). The availability of σ^E^ is controlled by antisigma factor, which sequesters σ^E^ to the membrane in an inactive state when membrane stress is absent. However, in the presence of stresses that lead to accumulation of misfolded proteins in the periplasm, a proteolytic cascade is initiated, releasing σ^E^ from antisigma factor. Free σ^E^ recognizes specific promoters and initiates transcription with core RNA polymerase (Rowley et al., 2006; Osterberg et al., 2011).

Using molecular genetic approaches and DNA microarray analyses, the σ^E^ regulon has been extensively studied in *E. coli* (Dartigalongue et al., 2001; Rezuchova et al., 2003; Kabir et al., 2005; Rhodius et al., 2006). However, the σ^E^ regulon obtained from *E. coli* is not directly applicable to *Salmonella* because σ^E^ plays distinct roles in these two bacteria. For instance, σ^E^ is required for viability in *E. coli*, while in *Salmonella*, it is essential for resisting reactive oxygen species, antimicrobial peptides, acid stress, and likely additional virulence related functions (Bang et al., 2005; Crouch et al., 2005; Muller et al., 2009). One of the *rpoE* promoters, rpoEp3, is more strongly induced by cold shock than by heat shock in *Salmonella*, but not in *E. coli*, reflecting the functional variation of σ^E^ in combating different stresses in these two organisms (Miticka et al., 2003). The *Salmonella* σ^E^ regulon has been studied using an *E. coli* two-plasmid screening system and by microarrays, however, the growth conditions exploited by previous studies did not discriminate different stages of *Salmonella* infection (Skovierova et al., 2006; Yoon et al., 2009).

Compared to other regulators important for *Salmonella* virulence (e.g., *fruR, ssrAB, slyA, crp, rpoS*, etc.), an *rpoE* mutant is the most sensitive to the intracellular environment. Specifically, the LD_50_ of an *rpoE* mutant in BALB/c mice by intraperitoneal infection is greater than 10^6^ CFU, whereas that of the parent strain is 1–2 CFU. Only a few, if any, viable *rpoE* mutants are recovered from primary macrophages after 30 min of infection (Yoon et al., 2009). It is unclear why the absence of *rpoE* in *Salmonella* has such an extreme phenotype during intracellular growth. Previous studies have shown that hundreds of genes were regulated by σ^E^, including genes in the *Salmonella* Pathogenicity Island 2 (SPI-2) (Osborne and Coombes, 2009; Yoon et al., 2009). SPI-2 genes encode components of a type III secretion system (TTSS) and its associated effectors, which are required for intracellular survival. The expression of SPI-2 genes is tightly regulated temporally and spatially. Without induction (e.g., carbon limitation, low concentrations of Mg^2+^ or Ca^2+^, and acidic pH), SPI-2 is bound by nucleoid-associated protein H-NS to silence transcription and avoid detrimental consequences of inappropriate expression. Inside professional phagocytic cells, SPI-2 expression is induced by the two-component systems PhoP/PhoQ, OmpR/EnvZ, and SsrA/SsrB (Lee et al., 2000; Garmendia et al., 2003; Bijlsma and Groisman, 2005).

To obtain a more complete picture of the σ^E^ regulatory network in *Salmonella*, in this paper we analyzed the transcriptional profile of σ^E^ by microarray on wild-type (WT) and *ΔrpoE* mutant cultured under three conditions: in nutrient-rich Luria-Bertani (LB) broth to log phase, in acidic minimal medium (LPM) for 4 h to mimic early intracellular infection, and in LPM for 20 h to mimic late intracellular infection. We established that a large number of *Salmonella* genes involving various functional categories are regulated by σ^E^, both directly and indirectly. Notably, we found that σ^E^ up-regulates SPI-2 gene expression through different mechanisms at different stages of infection: by increasing the transcription of SPI-2 activator *ssrB* in early stage, and by decreasing the transcription of SPI-2 repressor *hns* in late stage. σ^E^ can also counter the silencing of H-NS on SPI-2 genes.

## Materials and methods

### Bacterial strains and growth conditions

*Salmonella* STM ATCC 14028s was used as the parent strain in this study. The *rpoE*-deletion strain (*ΔrpoE*) was constructed using λ red recombination system as described (Yoon et al., 2009). Bacteria were grown under 3 different conditions to cover expression of a large number of *Salmonella* genes. They were: in Luria-Bertani (LB) medium to log phase (OD_600_ = 0.5), in pH 5.8, low phosphate, low magnesium-containing medium (LPM) for 4 h (OD_600_ ~ 0.5) or 20 h (OD_600_ ~ 1.0) (for the LPM culture, bacteria were grown in LB to stationary phase, washed twice in LPM, and resuspended in LPM at 1:10 dilution for an additional 4 h or 20 h) (Niemann et al., 2011). The latter two conditions partially mimic the intracellular environment of the *Salmonella*-containing vacuole and represent the early and late stage *Salmonella* infection. All the bacterial cultures were grown in triplicate. For microarray analysis 3 ml of culture was centrifuged, pellet was collected and treated with RNAlater (Ambion), then stored at -20°C prior to processing. The plasmid (pASK- H-NS -3xFLAG) expressing H-NS was constructed by cloning a DNA fragment containing coding sequence of *hns* on pASK-3xFLAG (constructed on pASK-IBA33plus by Hyunjin Yoon) via *Eco*RI and *Avr*II. Primers used in the PCR amplification of *hns* are shown in Table S1.

### Expression and purification of recombinant RpoE and H-NS

*S*. Typhimurium 14028s *rpoE* and *hns* genes were cloned into the plasmid pET200/D-TOPO (Invitrogen) using directional TOPO cloning method following the vendor's instructions. The genes were inserted downstream of the hexahistidine tag coding sequence under an isopropyl-1-thio-3/4-D- galactopyranoside (IPTG)-inducible T7 promoter. The bacterial expression constructs were confirmed by sequencing and transformed into BL21 (DE3) *E. coli* strain (Invitrogen). Transformed bacteria were grown in LB broth containing 60 μg/ml kanamycin to mid-log phase. IPTG was added to a final concentration of 1 mM and the incubation was allowed to proceed for 3 h at 37°C. Bacteria were harvested by centrifugation, stored at -80°C, and lysed in lysis buffer (10 mM Tris-HCl pH 7.5, 100 mM NaCl, 1 mM EDTA) supplemented with fresh protease inhibitor cocktail (Sigma) and 1 mg/ml lysozyme for 30 min on ice. Subsequently, the lysate was sonicated and clarified by centrifugation at 10,000 × g for 10 min at 4°C. The supernatant was incubated with HisPur™ Cobalt resin (Pierce) in batch and the beads were washed extensively with Wash buffer (50 mM sodium phosphate, 300 mM NaCl, 10 mM imidazole; pH 7.4). Bound proteins were eluted with Elution buffer (50 mM sodium phosphate, 300 mM NaCl, 150 mM imidazole; pH 7.4). Elution buffer was replaced with exchange buffer (50 mM Tris-HCl pH 8.0, 1% SDS, 1 mM EDTA, 10% glycerol) using an Amicon Ultra-4 Centrifugal filter unit (millipore) with a molecular weight cutoff of 3000 Da. A small portion of the eluted protein was withdrawn and subjected to immunoblot to confirm that RpoE and H-NS were expressed at the correct size. The remaining eluted protein sample was separated on SDS-PAGE, and stained with Coomassie blue. The single gel bands at the size of recombinant RpoE and H-NS (His tagged) were excised and sent to Pacific Immunology Corp. (Ramona, CA) for polyclonal antisera production.

### Purification of RpoE and H-NS antisera

The RpoE and H-NS antisera generated in rabbits were purified by affinity chromatography. Briefly, the purified recombinant RpoE or H-NS in coupling buffer (500 mM NaCl, 100 mM NaHCO_3_, pH 8.0) was mixed with activated CH Sepharose 4B (GE Healthcare) at 1:1 ratio and rotated for 4 h at 4°C. After washing away excess antigens, the remaining active groups on sepharose was blocked with 0.1 M Tris-HCl, then washed with buffers of alternating pH for 5 cycles. Each cycle consisted of a wash with 100 mM acetic acid/sodium acetate, pH 4.0 containing 500 mM NaCl followed by a wash with 100 mM Tris-HCl buffer pH 8.0 containing 500 mM NaCl. The RpoE or H-NS antisera were loaded onto antigen-coupled CH Sepharose 4B in a chromatography column (Bio-Rad). After sequential washes with PBS and lithium chloride solution (1 M LiCl, 150 mM NaCl, 0.5% Nonidet P-40, 10 mM Tris-HCl, pH 8) the column was eluted with 200 mM glycine pH 2 in 10 ml fractions by the addition of 10× PBS and 1% BSA. The pH of the eluates was adjusted to 7 with 5 N NaOH. Fractions containing the purified anti-RpoE and anti-H-NS antibodies as judged by SDS-PAGE were frozen at −20°C. Protein concentration determination was performed according to modified Lowry method using bovine serum albumin (BSA) as reference protein (Sandermann and Strominger, 1972).

### Microarray analysis

For each of the three experimental conditions (LB, LPM 4 h and LPM 20 h), we identified genes that were differentially expressed between the WT and *ΔrpoE* strains of *Salmonella*. The samples were assayed to the *Salmonella* Typhimurium/Typhi microarray (version 8), a two-channel spotted array (70-mer probes) designed by the Pathogen Functional Genomics Resource Center at the J. Craig Venter Institute (JCVI). The analysis consisted of quantifying spot intensities, handling low quality probes, correcting for background intensities, imputing missing values, summarizing replicate probes, normalizing the summarized intensities, and finally, finding differentially expressed genes.

First, we calculated a single, background-corrected intensity for the probes (spots) on each of our arrays. Using the scanned array image, we quantified the probe intensities using the Spotfinder tool from the TM4 Microarray Software Suite (Saeed et al., 2003, 2006), giving us an MEV file for each array. To load and manipulate the intensity data in the MEV files, we used Bioconductor's *limma* package (Gentleman et al., 2004; Smyth, 2005) We subtracted the background intensities from the foreground, giving us a background-corrected intensity for the probes on each array. If, after this step a probe had a negative intensity, we ignored it treating it as a missing value. We then identified and removed any replicate samples that did not have at least a 0.7 correlation with other replicates. For WT strain there were 13, 12, and 7 replicates that passed this array-level QC step in LB, LPM 4 h, and LPM 20 h conditions, respectively. For the *rpoE*-deletion strain, the corresponding numbers were 2, 4, and 2.

Next, we summarized replicate probe intensities into a single, normalized expression value for each gene. Before summarization, we imputed missing values using a k- nearest neighbors approach, as implemented in Bioconductor's *impute* package (Gentleman et al., 2004; Hastie et al.^1^). We then summarized the replicate intensities (there were two identical probes per gene) by calculating their mean. We finally normalized all of the mutant and WT expression values using quantile normalization, as implemented in the *normalize.quantiles* function of the *preprocessCore* R package (Bolstad et al., 2003; Bolstad^2^). We performed a separate normalization for each of the three experimental conditions.

Using the normalized expression values, we next identified differentially regulated genes between the Δσ^E^ and WT strains. Since our sample size for the knockouts was small, we decided to use the methodology described by Smyth et al., which involves using a moderated t-statistic that is more reliable for a small number of arrays (Smyth, 2004). The differential expression analysis was performed using functions available in the *limma* package. All microarray data is deposited on the Gene Expression Omnibus (GEO) at the National Center for Biotechnology Information (NCBI), accession numbers GSE25441 and GSE26755.

### Chromatin immunoprecipitation

*Salmonellla* 14028s grown in LB to log phase, or in LPM for 4 h or 20 h were cross-linked by 1% formaldehyde at room temperature for 25 min, then quenched using 125 mM glycine for an additional 5 min of incubation at room temperature. After cell lysis and sonication, cell debris was removed by centrifugation at 13,000 rpm for 10 min at 4°C, and the supernatant was collected for the immunoprecipitation. The supernatant was split into two samples. One was mixed with affinity purified rabbit anti-RpoE antibody to immunoprecipitate σ^E^–DNA complex, and the other sample was mixed with rabbit monoclonal antibody to GFP as the control (normal rabbit serum contains anti-*Salmonella* antibody so that was not used as control). They were next incubated at 4°C overnight, and 50 μl of the Dynabead M-280 sheep anti-rabbit IgG (Invitrogen) was added into the mixture. After 6 h of incubation at 4°C with rotating, the beads were washed with a serials of stringent buffers (Cho et al., 2008). Beads were resuspended in 200 μl of elution buffer (50 mM Tris-HCl at pH 8.0, 10 mM EDTA, and 1% SDS) and incubated at 65°C overnight to reverse the cross-linking. The supernatant was incubated with 1 μl of RNaseA (QIAGEN) for 2 h at 37°C to remove RNAs, followed by incubation with 4 μl of proteinase K solution (Invitrogen) for 2 h at 55°C to remove proteins. The sample was then purified with a PCR purification kit (QIAGEN). Gene-specific quantitative PCR was carried out using the DNA samples.

### Quantitative RT-PCR analysis

Total RNA was isolated using RNAlater Solution (Ambion), RNeasy mini kit (Qiagen), and DNase to remove residual chromosomal DNA (Qiagen) according to manufacturer's instructions. RNA concentration was measured by NanoDrop ND-1000 spectrophotometer (NanoDrop Technologies, Inc.). cDNA was synthesized using the iScript cDNA synthesis kit (Bio Rad) and cDNA corresponding to 10 ng of input RNA was used as template for real-time reaction containing Power SYBR green (Applied Biosystems) and gene-specific primers. The primers were designed with Primer Express 3.0 software and tested for their amplification efficiencies (Table S2). The *gyrB* gene, encoding for the B subunit of the DNA gyrase, was utilized as endogenous control. The RT-PCR reactions were carried out at 95°C for 10 min, 95°C for 15 s and 60°C for 1 min for 40 cycles (ABI 7700, Applied Biosystems). The expression ratio of each gene was the average from three independent RNA samples and was normalized to the level of *gyrB*.

### Immunoblot analysis

The WT strain containing pASK-H-NS-3FLAG plasmid was grown in LPM for 20 h in the presence or absence of anhydrotetracycline (AHT) for 4 h. The cells were washed and ~5 × 10^7^ colony-forming units were pelleted and re-suspended in Laemmli sample buffer, boiled for 5 min, and then separated on SDS-PAGE. Proteins on the gel were then transferred to polyvinylidene difluoride (PVDF) membranes (Millipore). After blocking in Tris-buffered saline (TBS) plus 5% non-fat dry milk for 1 h, membranes were probed with anti-FLAG monoclonal antibody (Sigma). Membranes were washed and probed with secondary antibody (anti-mouse IgG conjugated with peroxidase) (Sigma). The immune complexes were detected via chemiluminescence using Western Lightning™ (PerkinElmer), and then exposed to XAR Biofilm (Kodak). For immunoblot on H-NS, WT and *ΔrpoE* strains were grown in LB to log phase, or in LPM for 4 h or 20 h. Affinity purified rabbit anti-H-NS antibody was used as primary antibody, and goat anti-rabbit (IgG) antibody conjugated to horseradish peroxidase (Cell Signaling Technology) was used as the secondary antibody. In both cases, DnaK was probed at the same time as loading control.

## Results

### Identification of σ^E^-dependent genes by transcriptional profiling

**Figure 1:**
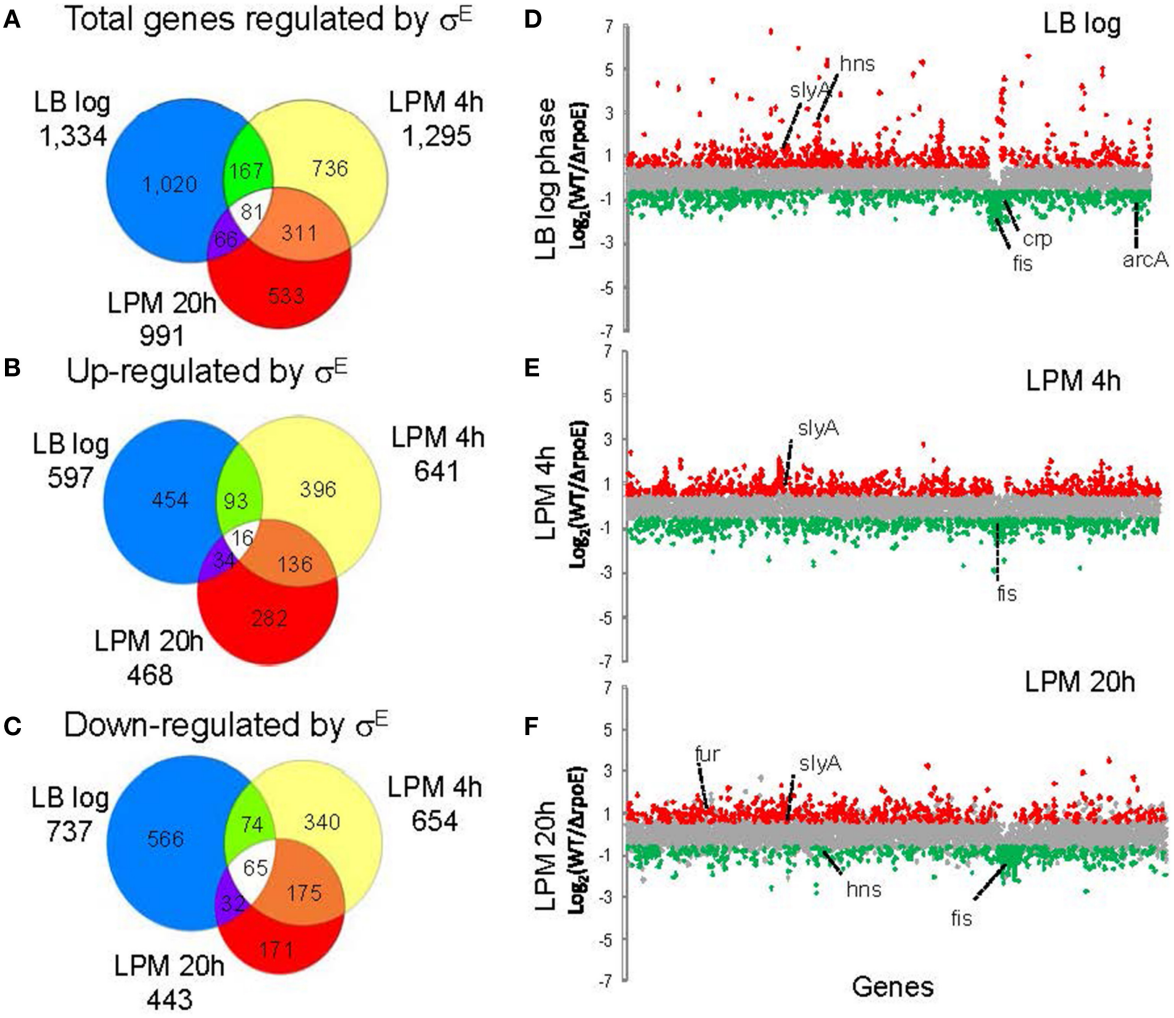
**Overview of gene expression regulated by σ^E^ in *Salmonella* Typhimurium cultured under three growth conditions**. *Salmonella* WT and *rpoE*-deletion strains were grown in LB medium to log phase or in acidic minimal medium (LPM) for 4 h or 20 h in triplicate. Total RNA was isolated and analyzed by the *Salmonella* Typhimurium/Typhi microarray (version 8). The Venn diagrams show overlaps of total genes regulated by σ^E^ **(A)**, genes up-regulated by σ^E^ **(B)** and genes down-regulated by σ^E^ **(C)** in the three growth conditions. The charts show differential gene transcription regulated by σ^E^ in LB log phase **(D)**, LPM 4 h condition **(E)** and LPM 20 h condition **(F)**. Each dot represents one gene of the *Salmonella* 14028s genome with the x-axis showing gene order in relation to the gene location on chromosome, and the y-axis showing log_2_-based fold changes of transcript of WT vs. *rpoE*-deletion strains. Genes activated by σ^E^ are red, while genes repressed by σ^E^ are green. Major global regulators regulated by σ^E^ are labeled.

To study the global regulatory effect of σ^E^ on *Salmonella* gene expression, WT (14028s) and *ΔrpoE* strains were grown in nutrient-rich LB broth to log phase, or in LPM for 4 h and 20 h to mimic early and late intracellular infection, respectively. *Salmonella* Typhimurium/Typhi microarray designed by the Pathogen Functional Genomics Resource Center at the J. Craig Venter Institute (JCVI) was used to analyze the transcriptional changes under each condition. Gene expression values were calculated as the log_2_ ratio of fold change of *rpoE* mutant to WT (Table S2), and based on positive or negative value of the ratio, the genes affected by σ^E^ are described as “activated (up-regulated)” vs. “repressed (down-regulated),” which does not necessarily imply a direct regulatory activity. In LB log phase, a total of 1334 genes were differentially expressed (*p* ≤ 0.05 and fold change = 1.5); in the LPM 4 h condition, 1295 genes were differentially expressed; in the LPM 20 h condition, 911 genes were differentially expressed. In total, 58% (2533 genes) of all 4355 *Salmonella* genes measured by the microarray were differentially expressed in at least one of the three conditions (i.e., their expression was σ^E^-dependent) (Figure 1A and Table S2). In each of the three conditions, the number of genes that σ^E^ activated and repressed was very similar, especially within the LPM 4 h and 20 h conditions (Figures 1B,C). Sigma factors are generally associated with positive regulation of gene expression, but because microarrays cannot discriminate between direct and indirect regulation, we expected and observed both positive and negative regulation. There were 81 genes, belonging to miscellaneous functional categories, which exhibited the same regulatory trend across all three conditions. Sixteen of these genes were up-regulated by σ^E^ in all 3 *in vitro* conditions, while 65 were down-regulated (Figures 1B,C and Table S3). To explain why σ^E^ has such a widespread effect on gene expression, we investigated the influence of σ^E^ on other *Salmonella* regulators. Approximately 40% of the known/predicted regulators (168 out of 411) were transcriptionally regulated by σ^E^ in at least one condition (Table S4). This group included most of the global regulators and many were differentially affected under the different growth conditions including Crp, Fis, ArcA, SlyA, Fur, and H-NS (Figures 1D–F). The regulation of these regulators by σ^E^ either directly or indirectly contributes to the complex regulatory network at different stages of *Salmonella* infection.

**Figure 2:**
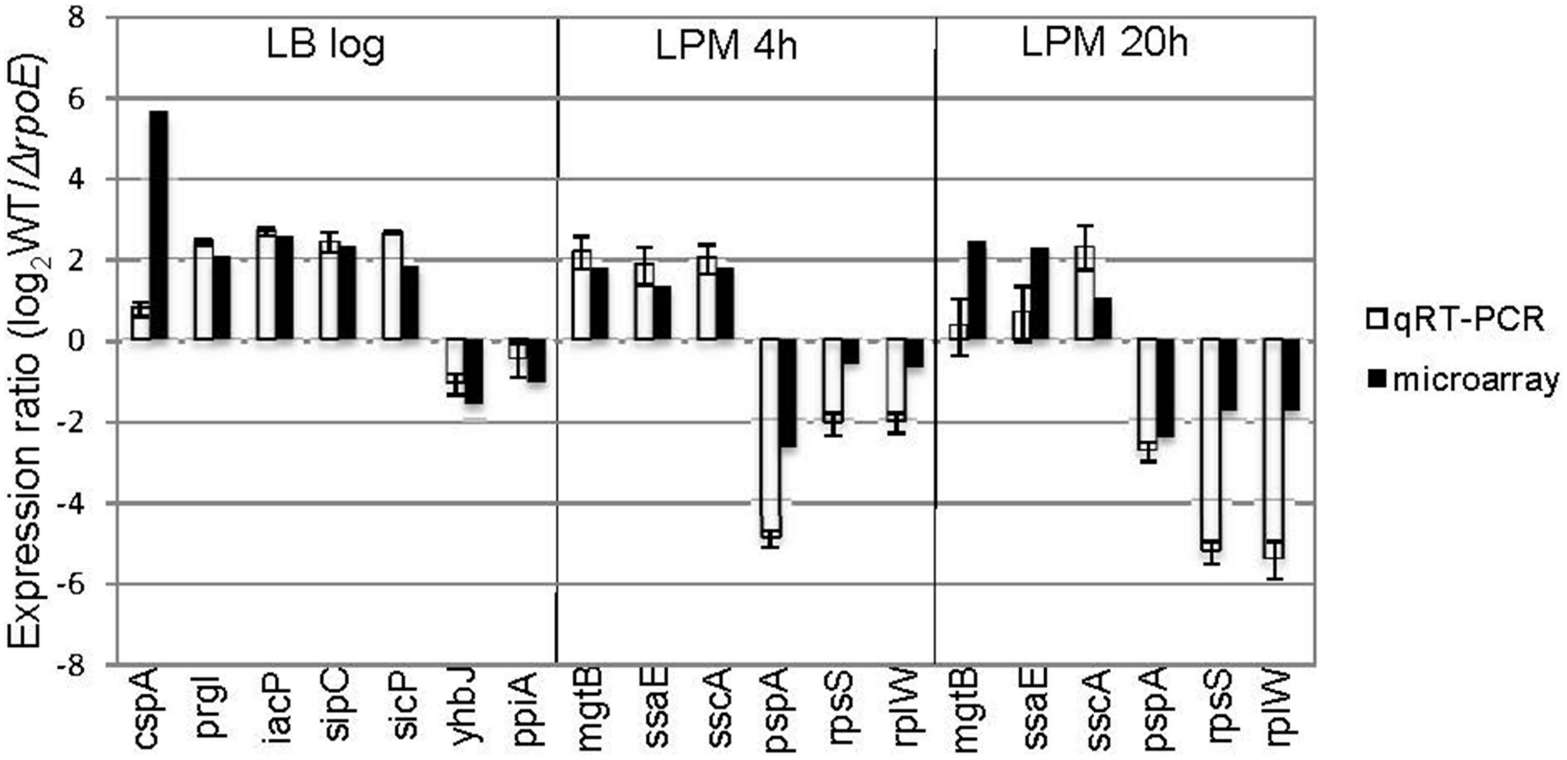
**Validation of microarray results by qRT-PCR**. Groups of genes both up- and down-regulated by σ^E^ were selected from three growth conditions (LB log, LPM 4 h, and LPM 20 h) based on microarray results, and validated by qRT-PCR using primers designed inside those genes. The results are plotted on a log_2_-scale comparing WT strain to *rpoE*-deletion strains. Values are normalized with *gyrB* mRNA levels and represent the average of RNA prepared from independent biological triplicates.

To further validate the microarray results, we conducted qRT-PCR on 13 genes that the microarrays identified as up- or down-regulated; in each case, qRT-PCR confirmed the microarray results (Figure 2). Moreover, out of the 62 σ^E^-dependent *Salmonella* genes previously identified by Skovierova et al., 44 (71%) of them were in agreement with our findings, including *rseA, rpoH, fusA, htrA, recB, eno, tolA, apl, yggT* (Skovierova et al., 2006).

### Functional categories and groups of genes regulated by σ^E^

The genes regulated by σ^E^ were involved in a broad spectrum of cellular functions, and classified into 19 categories according to JCVI annotation (Table 1). Comparing the genome annotation to the genes measured by microarray, our experiments covered 92–99% of each category. For the LB log phase, when cells are actively dividing, *energy metabolism* and *protein synthesis* were the two most abundant functional categories associated with up-regulated genes, whereas *transport and binding* and *cell envelope* functional categories were the most represented among down-regulated genes. For the LPM 20 h condition, where cells were exposed to significant stress, we observed the opposite scenario. Here, genes involved in *energy metabolism* and *protein synthesis* were mostly down-regulated by σ^E^, while genes encoding *transport and binding* and *cell envelope* proteins were up-regulated. For the LPM 4 h condition, the *cell envelope* category was most represented for up-regulated genes (the second most representative category of up-regulated genes at LPM 20 h), while *transport and binding* and *energy metabolism* was most represented for down-regulated genes. Across the three growth conditions, *cell envelope, energy metabolism*, and *transport and binding* functions were the top three categories of σ^E^-regulated genes.

**Table 1:** **JCVI functional categories of microarray results**.

**Functional Categories**	**Total from genome**	**Total measured by microarray**	**Up-regulated by σ^E^**			**Down-regulated by σ^E^**		
			**LB log**	**LPM 4 h**	**LPM 20 h**	**LB log**	**LPM 4 h**	**LPM 20 h**
Amino acid biosynthesis	130	125	17	13	11	16	20	19
Biosynthesis of cofactors, prosthetic groups, and carriers	167	163	24	5	8	16	38	21
Cell envelope	479	437	45	77	44	90	54	34
Cellular processes	289	263	43	55	35	54	33	18
Central intermediary metabolism	170	160	14	16	11	19	30	18
DNA metabolism	166	150	18	17	13	21	22	19
Energy metabolism	610	563	111	53	34	55	76	59
Fatty acid and phospholipid metabolism	80	77	6	8	6	9	17	7
Hypothetical proteins	78	65	11	11	9	12	7	3
Mobile and extrachromosomal element functions	250	155	8	33	18	36	10	8
Protein fate	191	181	26	38	29	27	24	16
Protein synthesis	375	313	61	37	28	44	54	54
Purines, pyrimidines, nucleosides, and nucleotides	81	80	8	3	2	10	20	7
Regulatory functions	305	268	22	46	29	44	42	22
Signal transduction	26	20	3	3	4	6	4	2
Transcription	57	54	10	4	3	4	7	6
Transport and binding proteins	628	579	51	48	53	100	81	49
Unclassified	332	267	39	56	37	36	37	23
Unknown function	670	614	69	66	73	105	95	58

**Figure 3:**
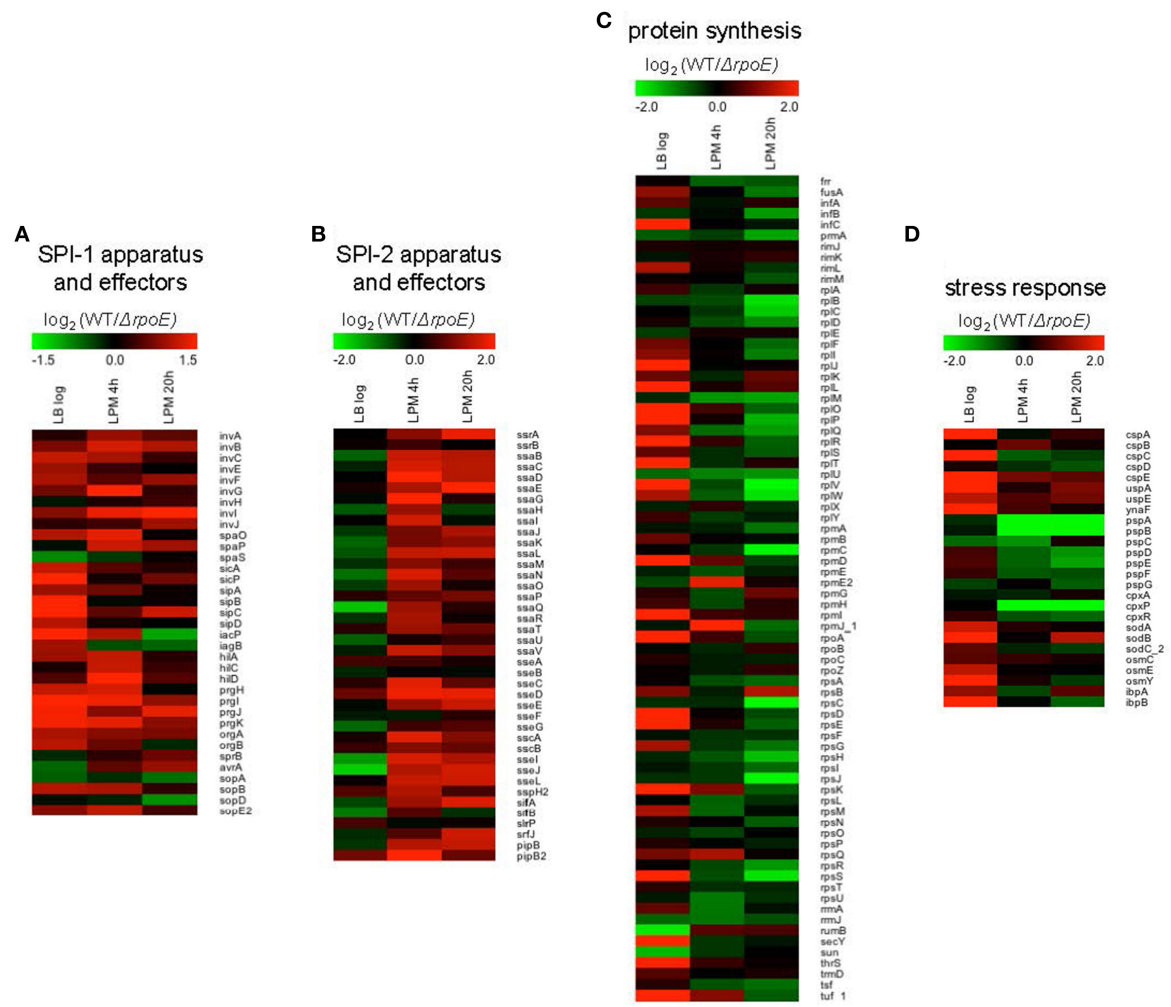
**Heat maps of four groups of genes that are differentially regulated by σ^E^ in *Salmonella* grown in LB to log phase, or in LPM for 4 h or 20 h**. Shown are genes involved in SPI-1 apparatus and effectors **(A)**, SPI-2 apparatus and effectors **(B)**, protein synthesis **(C)**, and stress response **(D)**. Red represents up-regulation of genes by σ^E^ while green represents down-regulation.

Notably, using the three growth conditions, we observed that σ^E^ differentially regulated four important groups of genes: (1) *SPI-1*; (2) *SPI-2*; (3) *protein synthesis*; and (4) *stress response*. SPI-1 genes were up-regulated by σ^E^ in LB log phase and in LPM 4 h condition, whereas in LPM 20 h condition, most of the SPI-1 genes were unaffected (Figure 3A). In contrast, SPI-2 genes were up-regulated by σ^E^ in LPM 4 h and 20 h conditions, but were unaffected by σ^E^ in LB log phase (Figure 3B). Our observations are consistent with previous report that σ^E^ activates the expression of most SPI-2 genes only when *Salmonella* are grown in minimal acidic media (Yoon et al., 2009). Genes associated with protein synthesis (e.g., translational initiation factors, protein translocation, and elongation) were up-regulated by σ^E^ in LB log phase, which contrasted with samples from the LPM 20 h condition, where they were down-regulated (Figure 3C). Consistent with previous findings that the phage shock protein (Psp) system and two-component regulatory system CpxR/CpxA compensate for the loss of σ^E^ function, we found that genes in the *psp* operon, including *pspA, pspB, pspD*, and *pspE*, were up-regulated in *ΔrpoE* under LPM 20 h condition when compared to WT (Figure 3D) (Connolly et al., 1997; Becker et al., 2005). The expression of *cpxP*, an indicator of the activation status of the CpxR/CpxA system, was also found to be elevated in *ΔrpoE* when compared to WT in LPM 4 h and 20 h samples (Figure 3D) (Kato et al., 2012). Moreover, σ^E^ up-regulated the cold-shock protein genes *cspA*, *cspC*, and *cspE*; the universal stress genes *uspA* and *ynaF*; the oxidative stress-response genes *sodA, sodB, and sodC_2*; the hyperosmotic stress genes *osmC, osmE, and osmY*; and the heat shock protein genes *ibpA* and *ibpB* under LB log phase condition. These results suggest that σ^E^ coordinates multiple stress response systems to achieve appropriate gene regulation.

### σ^E^ up-regulates SPI-2 gene expression by increasing *ssrB* transcription in the condition that mimics early infection

**Figure 4:**
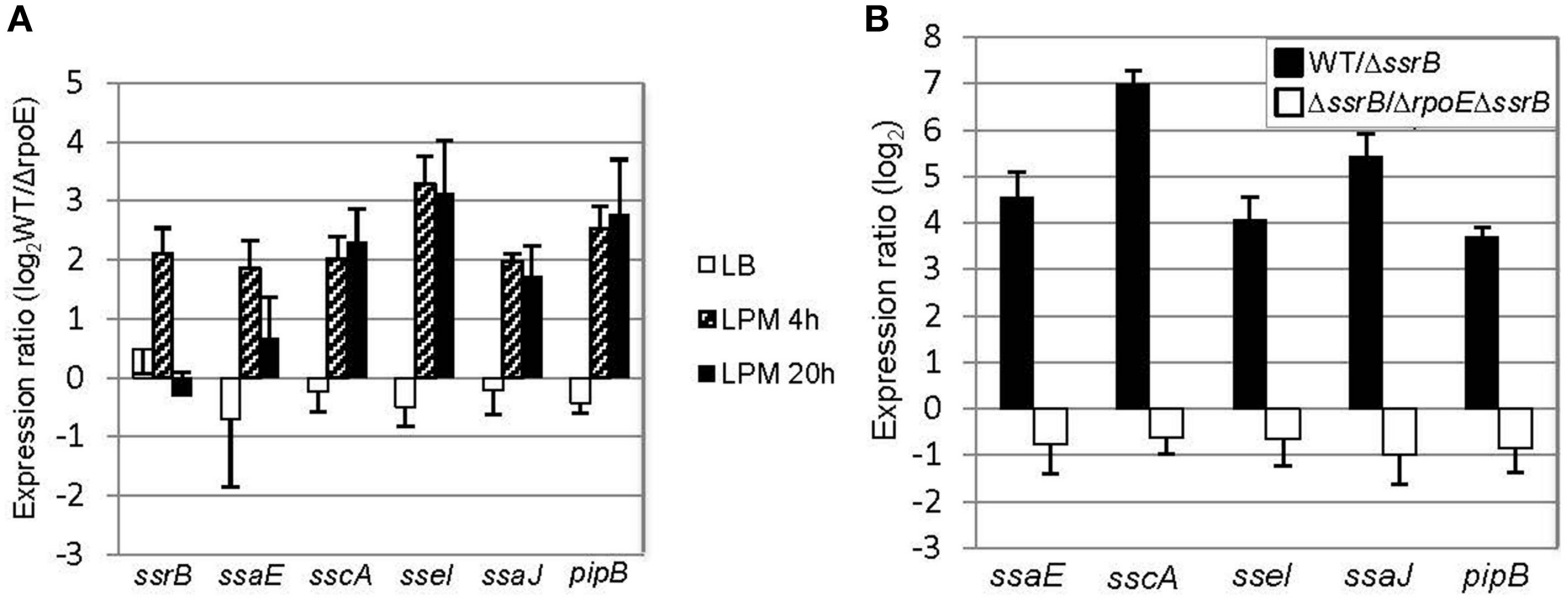
**σ^E^ up-regulates SPI-2 gene expression by increasing *ssrB* transcription under LPM 4 h condition. (A)** σ^E^ regulates SPI-2 gene expression differently in three growth conditions. The WT and *ΔrpoE* strains were grown in LB to log phase, or in LPM for 4 h or 20 h. SPI-2 gene expression was measured by qRT-PCR using *gyrB* as an internal control. Shown are expression ratios comparing SPI-2 gene level in WT vs. *ΔrpoE* strains prepared from independent biological triplicates. **(B)** The effects of SsrB and σ^E^ on SPI-2 gene expression in LPM 4 h condition. The WT, *ΔssrB*, and (*ΔssrB, ΔrpoE*) strains were grown in LPM for 4 h. The transcription of SPI-2 genes was measured by qRT-PCR. Expression ratio comparing WT to *ΔssrB* indicates that SsrB activates SPI-2 gene expression; Expression ratio comparing *ΔssrB* to (*ΔssrB, ΔrpoE*) indicates that the up-regulating effects of σ^E^ on SPI-2 gene expression are through SsrB in LPM 4 h condition.

Although σ^E^ has been found to regulate SPI-2 gene expression during infection-like conditions in previous studies, the mechanism of how it accomplishes this is still not clear (Osborne and Coombes, 2009; Yoon et al., 2009). To investigate how σ^E^ manipulates SPI-2 gene expression, we selected four genes within SPI-2 in different operons (*ssrB, ssaE, sscA*, and *ssaJ*) and two genes outside of SPI-2 (*sseI* and *pipB*) that encode the effectors secreted by the SPI-2 TSSS, and then compared their transcription in WT vs. *ΔrpoE* strain in LB log phase, LPM 4 h and LPM 20 h conditions (Figure 4A). We observed that σ^E^ up-regulated the expression of all the above genes in the condition that mimics early infection (LPM 4 h) (Figure 4A, patterned bars). Since *ssrB* encodes a general activator of SPI-2 genes, we investigated whether or not σ^E^ up-regulated SPI-2 gene expression in LPM 4 h condition by increasing *ssrB* transcription (Coombes et al., 2007; Yoon et al., 2009). We confirmed that the presence of SsrB increased the expression of the selected SPI-2 genes studied here using qRT-PCR, by comparing the expression ratio of these SPI-2 genes in WT vs. *ΔssrB* strains (Figure 4B, black bars). To eliminate the effects of SsrB on σ^E^–mediated SPI-2 gene expression, we constructed an *rpoE*/*ssrB* double mutant strain and compared *ssaE, sscA, sseI, ssaJ*, and *pipB* expression in *ΔssrB* vs. (*ΔrpoE, ΔssrB*). The expression ratios of the above 5 genes showed no significant difference between these two strains (Figure 4B, white bars), which means deletion of *ssrB* abolished the effects of σ^E^ on SPI-2 genes in LPM 4 h condition. Altogether, our results suggest that σ^E^ up-regulates *ssrB* transcription which results in increased SPI-2 gene expression in the condition that mimics early infection.

### σ^E^ up-regulates SPI-2 gene expression by repressing *hns* transcription in the condition that mimics late infection

**Figure 5:**
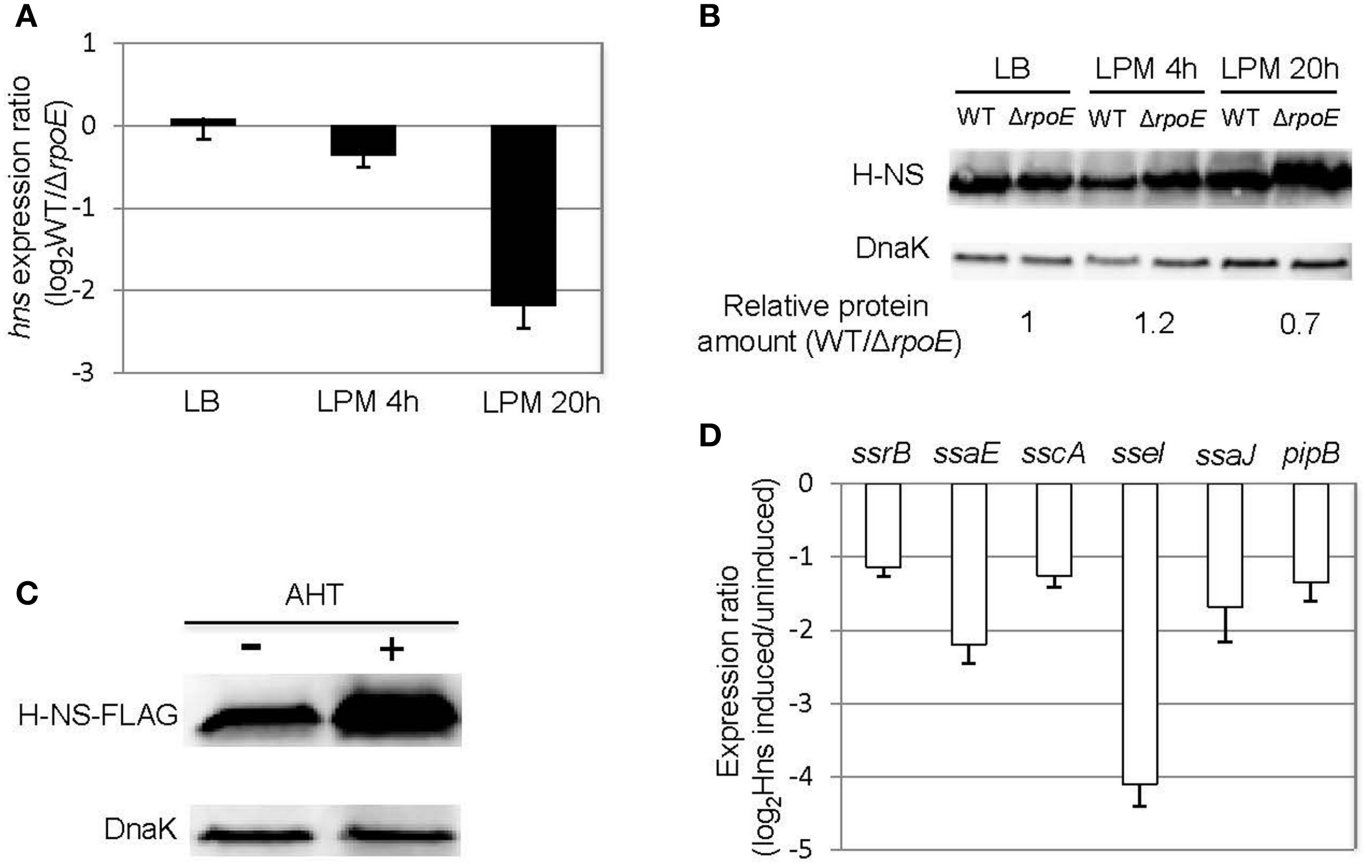
**σ^E^ up-regulates SPI-2 gene expression by repressing *hns* transcription under LPM 20 h condition. (A)** σ^E^ down-regulates *hns* expression in LPM 20 h condition. The WT and *ΔrpoE* strains were grown in LB to log phase, or in LPM for 4 h or 20 h in biological triplicates. The transcription of *hns* was measured by qRT-PCR using *gyrB* as an internal control. The expression ratio compares the level of *hns* in WT vs. *ΔrpoE* in each condition. **(B)** The effects of σ^E^ on H-NS expression in LB log phase, LPM 4 h, and LPM 20 h conditions. Western blots of H-NS in protein lysates from WT and *ΔrpoE* strains were generated using affinity purified rabbit anti-H-NS antibody. DnaK was used as loading control. For quantification, H-NS level in each strain under each condition was normalized to DnaK level, then relative protein amount (WT/Δ*rpoE*) for each condition was calculated. The ratios of protein amount are shown at the bottom. **(C)** Overexpression of H-NS through AHT induction. *Salmonella* 14028s strain was transformed with a plasmid containing FLAG-tagged H-NS (pASK-H-NS-3xFLAG) under AHT-inducible promoter. The bacteria were grown in LPM for 20 h in the presence or absence of AHT. The level of H-NS was detected by Western blot using monoclonal antibody to FLAG. **(D)** Overexpression of H-NS represses SPI-2 gene expression. The WT strain containing pASK-H-NS-3xFLAG plasmid was grown in LPM for 20 h with or without AHT induction. SPI-2 gene expression was measured by qRT-PCR. The results are presented as expression ratio comparing the strain in which H-NS is induced vs. un-induced on a logarithmic scale.

In the condition that mimics late infection (LPM 20 h), the expression of *ssrB* was unaffected by the presence of σ^E^ (Figure 4A), suggesting that the general activator SsrB was not causing the up-regulation of SPI-2 genes at this time point. We next considered whether a change to the transcription of general negative regulators might indirectly induce the expression of SPI-2 genes. We focused on the nucleoid-associated protein H-NS because it has been shown to be a general repressor of SPI-2 genes (Navarre et al., 2006). Under LPM 20 h condition, microarray data showed that σ^E^ repressed the expression of *hns* (Table S2), which was further confirmed with qRT-PCR (Figure 5A). Although other nucleoid-associated proteins, such as YdgT, Hha, and StpA, also recognize and selectively silence the expression of foreign DNA, none of them were down-regulated by σ^E^ (Table S2), and therefore were not further investigated. Western blot analysis indicated that σ^E^–mediated *hns* down-regulation was also reflected at the protein level in LPM 20 h condition (Figure 5B). The effects of H-NS on SPI-2 gene expression in LPM 20 h condition was examined by over-producing H-NS instead of deleting *hns*, as it is required for viability in *Salmonella*. Construction of a *Δhns* strain requires deletion of an extra gene (*rpoS* or *phoP*), which would complicate the analysis (Navarre et al., 2006). The WT strain was transformed with a plasmid containing a FLAG-tagged H-NS under a tetracycline-regulated promoter. As expected, H-NS was strongly induced by the addition of anhydrotetracycline (AHT) as visualized by western blot using anti-FLAG antibody (Figure 5C), and was further verified using H-NS specific rabbit polyclonal antibodies (data not shown). We measured the expression ratios of SPI-2 genes when H-NS was induced vs. non-induced and found that induction of H-NS resulted in 2–16 fold decrease of SPI-2 gene expression (Figure 5D). Therefore, under LPM 20 h condition, H-NS functions to repress SPI-2 gene expression. Since σ^E^ represses *hns* transcription under the same condition, collectively our results suggest that under the condition that mimics late infection, σ^E^ up-regulates SPI-2 expression by repressing *hns* transcription.

### σ^E^ counters the silencing of H-NS on SPI-2 gene expression

**Figure 6:**
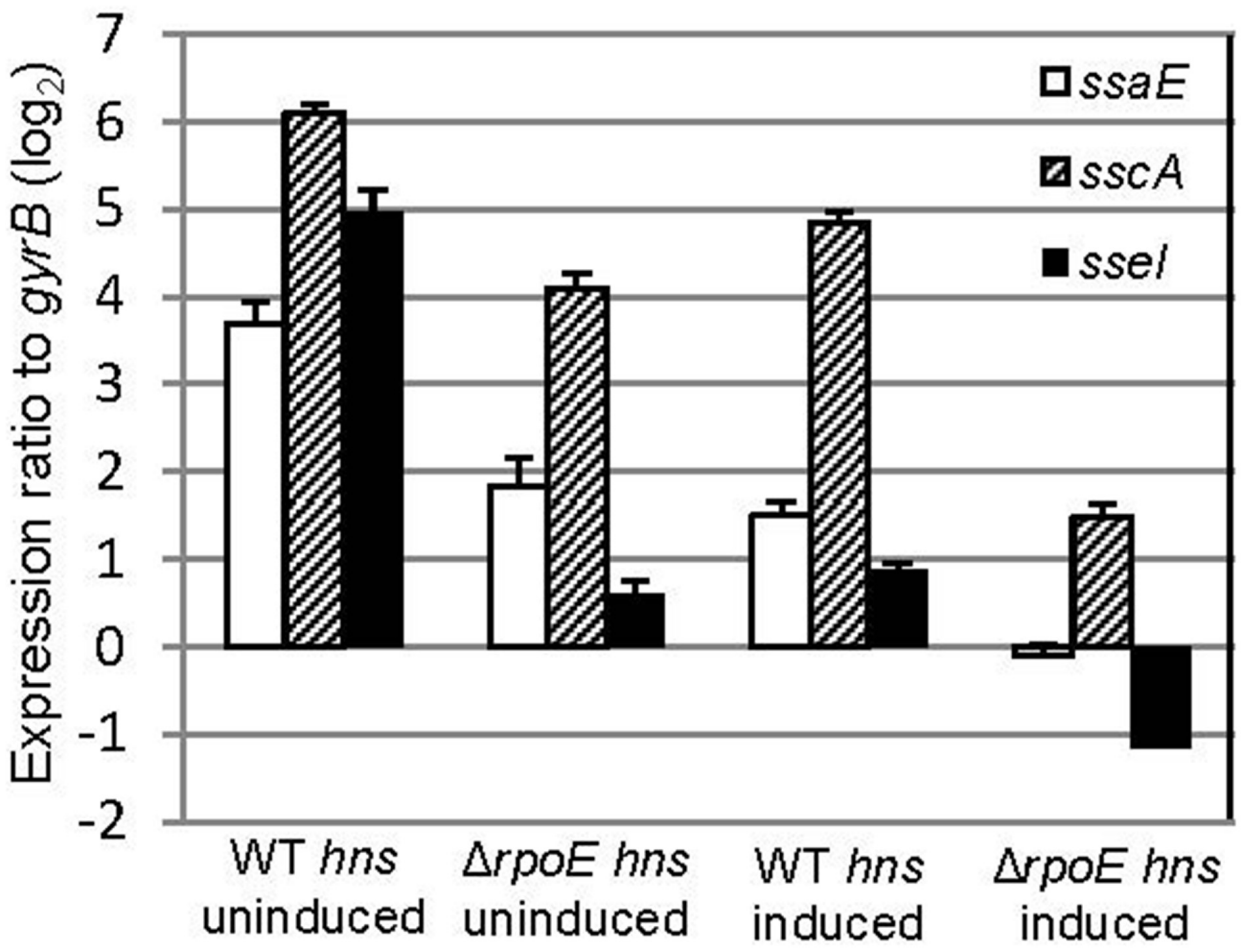
**σ^E^ counters the silencing of H-NS on SPI-2 gene expression**. The WT and *ΔrpoE* strain were transformed with plasmid pASK-H-NS-3xFLAG, and grown in LPM for 20 h in the presence or absence of AHT. SPI2 gene (*ssaE*, *sscA*, and *sseI*) expression were measured by qRT-PCR in each condition (triplicate biological samples) and standardized to *gyrB* in a logarithmic scale.

Several regulators (e.g., σ^S^, SsrB and SlyA) have been shown to be capable of relieving H-NS silencing of transcription (Mujacic and Baneyx, 2006; Perez et al., 2008; Walthers et al., 2011; Galagan et al., 2013). To investigate if σ^E^ can counter the H-NS silencing of SPI-2 gene expression, we transformed both WT and *ΔrpoE* strains with the AHT-inducible plasmid (pASK-H-NS-3xFLAG), which allowed us to change the expression of both *hns* and *rpoE* independently. The expression of SPI-2 apparatus (*ssaE, sscA*) and effector (*sseI*) genes were measured using qRT-PCR with samples generated from LPM 20 h conditions with and without AHT induction (Figure 6). Whether *hns* was induced or not, the expression of *ssaE, sscA*, and *sseI* were higher in WT than in *ΔrpoE* strain (comparing “WT *hns* uninduced” to “*ΔrpoE hns* uninduced,” or “WT *hns* induced” to “*ΔrpoE hns* induced”), which is consistent with results in Figure 4A. The overexpression of H-NS repressed SPI-2 gene expression (comparing “*ΔrpoE hns* uninduced” to “*ΔrpoE hns* induced”), however when σ^E^ was present, the repression of H-NS on SPI-2 was relieved (compare “WT *hns* induced” to “*ΔrpoE hns* induced”). These results suggest that σ^E^ is capable of countering the silencing of H-NS on SPI-2 genes.

### σ^E^ regulates *ssrB* and *hns* expression indirectly

**Figure 7:**
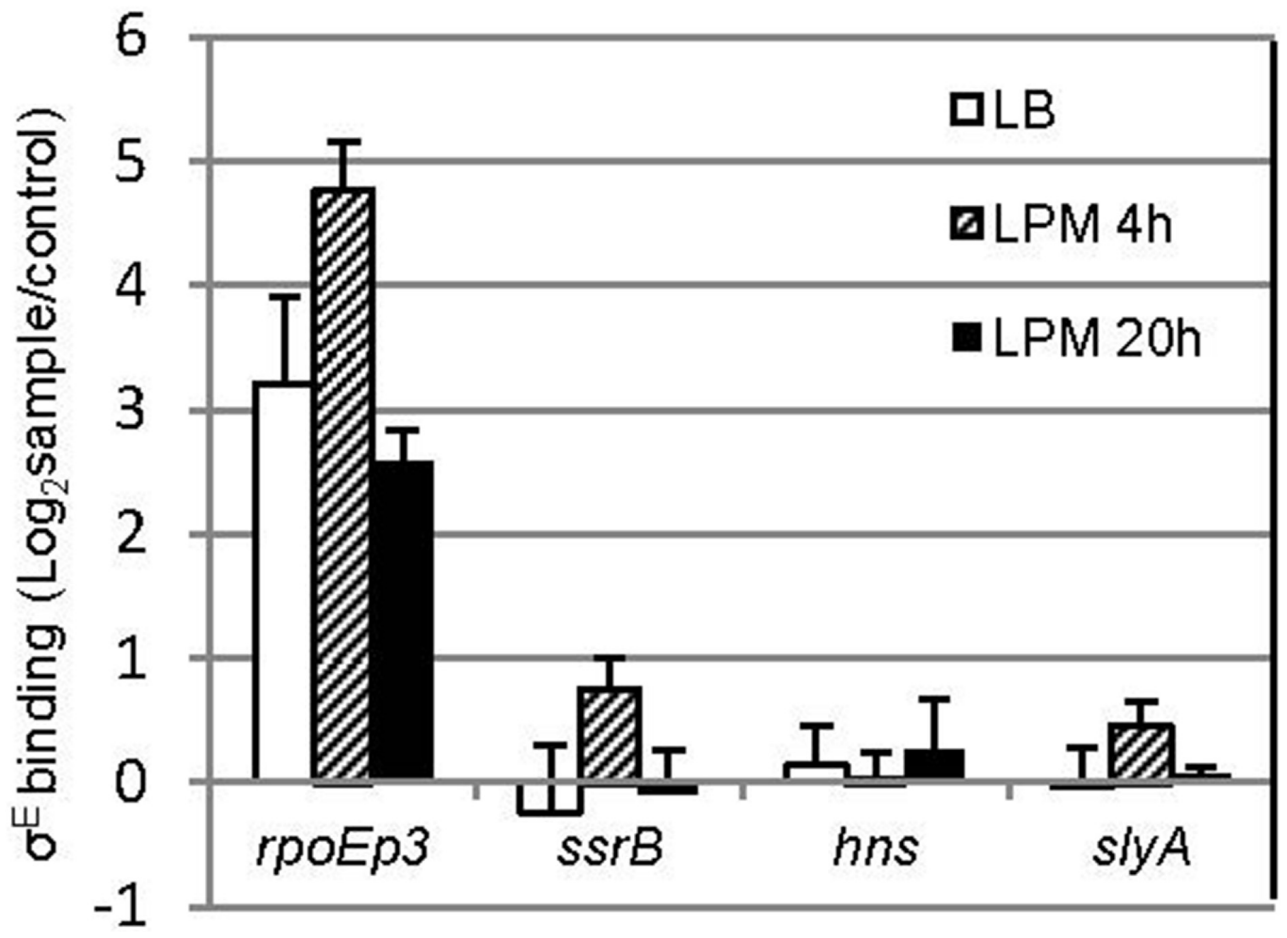
**σ^E^ does not bind to the promoter of *ssrB, hns*, and *slyA in vivo***. *Salmonellla* 14028s was grown in LB to log phase, or in LPM for 4 h or 20 h, crosslinked, sonicated, and immunoprecipitated with affinity purified rabbit anti-RpoE antibody (sample) or rabbit monoclonal antibody to GFP (control) to pull down σ^E^ interactors, after removal of proteins and RNAs, purified DNA was used as template for quantitative PCR with primers designed around the promoter region of each gene. The promoter 3 region of *rpoE* (rpoEp3) was used as a positive control. Shown are the binding ratios comparing σ^E^-specific vs. non-specific binding displayed in a logarithmic scale. The mean and S.D. values were obtained from independent biological triplicates.

σ^E^ activates transcription by recognizing a canonical binding motif at the promoter region (Skovierova et al., 2006; Osterberg et al., 2011). To investigate if *ssrB* and *hns* are directly regulated by σ^E^, we searched the -35 and -10 elements of *ssrB* and *hns* but failed to identify σ^E^–dependent promoter (data not shown). Since not all of the σ^E^-binding sites in *Salmonella* contain the specific motif (*in vivo* ChIP-seq, unpublished data), which is also true for other regulators (Galagan et al., 2013), we performed a chromatin immunoprecipitation combined with quantitative PCR assay (ChIP-qPCR) that compared the enrichment of target regions in pulldowns using anti-σ^E^ or control (anti-GFP) antibody (Figure 7). Compared to *rpoE* promoter 3, which is a known binding site of σ^E^, primers designed around the promoter region of *ssrB* and *hns* yielded no significant enrichment. Another general regulator of SPI-2 gene, SlyA, was also studied, and similarly did not exhibit elevated binding to σ^E^ than control. Our results suggest that there is no *in vivo* occupancy of *ssrB* and *hns* promoters by σ^E^, therefore, it is likely that σ^E^ indirectly regulates *ssrB* and *hns*, which up-regulates SPI-2 gene expression.

## Discussion

As an alternative sigma factor, σ^E^ functions to coordinate gene expression in response to extracytoplasmic stresses. It recognizes a “canonical sequence” at the promoter region of target genes and initiates transcription with core RNA polymerase (Skovierova et al., 2006; Osterberg et al., 2011). Compared to the genes reported to be regulated by σ^E^ in *Salmonella*, our study expanded the σ^E^ regulon enormously, partially because multiple growth conditions that mimic different stages of *Salmonella* infection were exploited (Skovierova et al., 2006; Yoon et al., 2009). A large number of genes regulated by σ^E^ are involved in various biological processes including cell envelope biosynthesis and degradation, energy metabolism, protein synthesis, transport and binding, as well as functions that are not known. Since the activation of SPI-2 genes is essential for *Salmonella* intracellular survival and our current and previous studies indicated that σ^E^ up-regulates SPI-2 gene expression under infection-like conditions, we further investigated how σ^E^ manipulates SPI-2 gene expression (Yoon et al., 2009). We found that σ^E^ indirectly regulates *ssrB* and *hns* transcription, also counters the silencing of H-NS on SPI-2 genes. Together with more than a 100 other *Salmonella* regulators transcriptionally regulated by σ^E^, it is likely the strong regulatory effects of σ^E^ may account for the extremely attenuated phenotype exhibited in *rpoE* null mutant.

Many regulators of *Salmonella* act on SPI-2 by regulating the two-component regulator SsrB and the MarR-type regulator, SlyA (Yoon et al., 2009). It is likely the effects of σ^E^ on SPI-2 transcription is mediated by SsrB because overexpression of SsrB, but not SlyA, can complement the decrease of SPI-2 expression as a result of *rpoE* deletion (Yoon et al., 2009). Therefore, in this study, we focused on the coordinated function of *ssrB* and σ^E^ on SPI-2 regulation without examining the involvement of SlyA (Figure 4A). We found that σ^E^ up-regulated SPI-2 gene expression through *ssrB* in early stages of infection, and through *hns* during late stages of infection. It is not known why *Salmonella* utilizes different mechanisms to meet its need in activating SPI-2 genes, yet in late stages of infection overall protein synthesis was down-regulated by σ^E^ (Figure 3C), consistent with its effect on *hns*. Moreover, neither *ssrB* nor *hns* was regulated by σ^E^ directly (Figure 7), on the contrary, both *ssrB* and *hns* contain σ^D^-recognizable promoters, consistent with previous findings (Kroger et al., 2012). Since σ^D^ has been shown to be directly regulated by σ^E^, we speculate that σ^E^ might manipulate *ssrB* and *hns* transcription through a σ^E^−σ^D^-*ssrB/hns* cascade (Skovierova et al., 2006). Alternatively, the cascade could be represented as σ^E^−σ^D^-*ompR/slyA/phoP*- *ssrB/hns* as OmpR, SlyA, and PhoP have been shown to directly regulate *ssrB* and all of their transcriptional start sites are associated with σ^D^ (Lee et al., 2000; Feng et al., 2003; Bijlsma and Groisman, 2005; Okada et al., 2007; Kroger et al., 2012). Further investigations are needed to verify the above hypotheses.

This is the first report that σ^E^ is capable of countering the silence of H-NS on SPI-2 genes (Figure 6). The SPI-2 genes of *Salmonella* are normally bound by H-NS, which is a barrier to transiting RNA polymerase. However, this transcriptional barrier is relatively weak (_~_7pN) and is easily overcome in conditions that induce SPI-2 expression (Fang and Rimsky, 2008). It has been reported that SsrB counters the silencing of H-NS on SPI-2 genes by displacing H-NS bound in its polymerization mode, and subsequently activates SPI-2 transcription (Walthers et al., 2007, 2011). In contrast, SlyA does not displace H-NS from the DNA, but remodels the H-NS-DNA nucleoprotein complex to recruit RNA polymerase and promotes PhoP-mediated gene transcription (Perez et al., 2008). σ^S^ counters the silencing of H-NS on selective genes that can be transcribed by both σ^D^ and σ^S^, since H-NS assembles nucleoprotein complexes with σ^D^ but not σ^S^ RNA polymerase holoenzyme, σ^S^ is able to escape H-NS trapping and the genes can be transcribed through σ^S^–dependent promoters (Shin et al., 2005; Typas et al., 2007). Whether σ^E^ exploits similar mechanisms as SsrB, SlyA, or σ^S^ or uses another mechanism to relieve H-NS silencing on SPI-2 genes is under investigation.

While entering into stationary phase is known to induce σ^E^, the actual signal that activates σ^E^ is the accumulation of misfolded outer membrane proteins within the periplasm, which occurs under a variety of conditions (Mecsas et al., 1993; Missiakas et al., 1996; Raivio and Silhavy, 1999). Here, we reported that a large amount of genes are regulated by σ^E^ in LB log phase (Figure 1), consistent with previous results (Kabir et al., 2005). Although the house-keeping sigma factor σ^D^ is considered to play the major role to maintain metabolism during exponential phase and is required for viability as expected, σ^E^ is also required for growth in both normal and stress conditions (De Las Penas et al., 1997). σ^E^ and σ^D^ recognize different binding motifs, however, both of them can activate multiple general regulators, through which the regulation effect is magnified (Hook-Barnard et al., 2006; Rhodius et al., 2006). We also found that alternative sigma factors function together to regulate stress response genes. For instance, the heat shock protein genes *ibpA* and *ibpB* are directly regulated by σ^H^, and indirectly, by the action of σ^E^ on σ^H^ (Figure 3D) (Nonaka et al., 2006; Skovierova et al., 2006). σ^S^ is the master regulator of stress response genes, and plays a dominant role in regulating hyperosmotic stress in *Salmonella* (Hengge-Aronis, 2002; McMeechan et al., 2007). We observed that the osmolarity stress genes *osmB, osmC*, and *osmY* are also regulated by σ^E^ (Figure 3D) (Bang et al., 2005). The co-regulation of gene expression by sigma factors benefits the pathogen by simultaneously inducing general and specific stress responses to many environmental factors, even if that resistance is not immediately required (Battesti et al., 2011).

In addition to up-regulating gene expression, σ^E^ was found to down-regulate a large number of genes in our study (Table S2). This phenomenon has been observed before in both *Salmonella* and *E. coli*, however at a much smaller scale (Bang et al., 2005; Kabir et al., 2005). The down-regulation of gene expression may not be a direct effect of σ^E^, rather an indirect repression through its downstream transcriptional regulators, or by binding to small non-coding RNAs (sRNAs), such as RybB and MicA, both of which have been found to function as global regulators (Papenfort et al., 2006; Gogol et al., 2011). Some of the genes repressed by MicA and RybB were also found to be down-regulated by σ^E^ in our study, such as *pal* and *gloA* (Table S2). Additionally, we found that σ^E^ regulates *hfq* expression in both nutrient-rich and infection-like conditions (Table S2). Hfq is a sRNA-binding protein that bridges sRNA to act on *trans*-encoded target mRNAs, therefore modulates the stability and translation of the target mRNAs (Vogel and Luisi, 2011). Both RybB and MicA are regulated by Hfq in repressing outer membrane protein expression. For instance, Hfq significantly enhances RybB binding to *ompC* and *ompD* mRNAs, facilitating the decay of *omp* mRNAs when the cell experiences extracytoplamic stress (Udekwu et al., 2005; Papenfort et al., 2006). Hfq controls the expression of at least 20% of all *Salmonella* genes directly or indirectly, and has a pleiotropic effect on *Salmonella* virulence through regulation of motility, outer membrane protein expression, invasion, and intracellular growth (Sittka et al., 2007, 2008; Ansong et al., 2009). As the list of sRNAs in *Salmonella* grows, these newly identified sRNAs may help to explain the global impact of σ^E^ on gene expression (Kroger et al., 2012).

By comparing the global transcription profiles of σ^E^ across three growth conditions, we established that σ^E^ coordinated the gene expression of *Salmonella* for proliferation and/or survival in response to various environments. The role of σ^E^ in global gene regulation and SPI-2 gene activation observed in this study explains why σ^E^ is required for systemic mouse infection, and shows that σ^E^ is the conductor of the *Salmonella* gene regulation orchestra, whether under stress or not.

### Conflict of interest statement

The authors declare that the research was conducted in the absence of any commercial or financial relationships that could be construed as a potential conflict of interest.

## Footnotes

^1^Hastie, T., Tibshirani, R., Narasimhan, B., and Chu, G. Impute: Impute: Imputation for Microarray Data. R package version 1.40.40.

^2^Bolstad, B. M. preprocessCore: A Collection of Pre-Processing Functions. R Package Version 1.28.20.

## Supplementary material

Table S1 **List of primers used in this study**.

Table S2 **List of genes regulated by σ^E^ under three growth conditions**.

Table S3 **List of genes up- and down-regulated by σ^E^ in all three growth conditions**.

Table S4 **List of regulators regulated by σ^E^ in at least one of the three growth conditions**.
