# Supplementary material for: Analysis of the Salmonella regulatory network suggests involvement of SsrB and H-NS in σE-regulated SPI-2 gene expression
Source: Front Microbiol. 2015 Feb 10;6:27. doi: 10.3389/fmicb.2015.00027 (PMC4322710; doi:10.3389/fmicb.2015.00027)
Supplement: Table S1 — List of primers used in this study. [file Table1.DOCX]

**Table S1.** List of primers used in this study.

| Primers | Sequences (5’ - end to 3’ - end) | Purpose |
| --- | --- | --- |
| cspA-F1 | GGACGAAGGTCAGAAAGTTTCCT | qRT-PCR |
| cspA-R1 | GCTGCCGGGCCCTTAG | qRT-PCR |
| prgI-F1 | GAGGCGCTGGATAAATTAGCA | qRT-PCR |
| prgI-R1  iacP-F1  iacP-R1  sipC-F1  sipC-R1  sicP-F1  sicP-R1  yhbJ-F1  yhbJ-R1  ppiA-F1  ppiA-R1  mgtB-F1  mgtB-R1  ssaE-F1  ssaE-R1  sscA-F1  sscA-R1  pspA-F1  pspA-R1  rpsS-F1  rpsS-R1  rplW-F1  rplW-R1  sseI-F1  sseI-R1  ssrB-F1  ssrB-R1  ssaJ-F1  ssaJ-R1  pipB-F1  pipB-R1  hns-F1  hns-R1  rpoEp3-F1  rpoEp3-R1  ssrB-F1  ssrB-R1  hns-F1  hns-R1  slyA-F1  slyA-R1  hns-F  hns-R | ACTCTGATACGCCGCCAGTAG  GGAGTTTGACATTAGTTGCAATGAA  CGGCATATATCCGCAAAGGT  GCGCGCAAGATGCAGAT  CGACCGTGACCGAGTTCTTC  CGCCTGTTTGTGGCGATT  TCGCAGCCAGTTCACCATTA  ACGCTCACGTGCGGATCT  CCAGCTCATGGACGGACAT  TCCGCCGGGAATATTGAG  CACAGAAACCGGCGCTTT  TCTGGCAGGAGTTTCGCACTA  GGTGGTGCGAACCATCTTTT  CCGCAGCAATATCAGCAAAA  AAGTGCGCTGTTATGGTAACGA  GGCTCGCTGCGTATGTTGTT  GCCGGCGAATTCTTTTACCT  ATCAGGCGGCGAGTTCTTC  CCAGTTTGCCGCTGTCAAG  TCGGTCACAAACTGGGTGAA  GCGTGGCCGCGATAAG  AAAGCGTCTACTGCGATGGAA  GTCGCGTCTTTAGCAACTTTGA  GATACCCCCCCTGAAATGAGTT  GTGACAAATCGTCCAGATGCA  CGCAGGTGCTAATGGCTATG  TTTGCAATGCCGCTAACAGA  TGTCGAGCAGTCGCAGTTTATTA  TGCCTATGCGGATAACCGTTA  AACCGAGGATGGTTTTCTGAAC  TTCCCCCTGGAAATCTTTATGA  GGCATTGACCCGAATGAACT  CGTTTAGCTTTGGTACCGGATT  GCTTATGGAGTGGCGTTTCG  GAGGTAATGTCTCCCCAAACCA  GGCAGACTGAATTGGTATGCTATG  GCGATGATTTTCCCCATTTTT  GCTCAACAAACCACCCCAAT  ACGGATGTTGTTCAGAATTTTAAGTG  GCAATCCTGTGGCGTTGAGT  GCATCGATGTCTGACGCAAA  GATCGAATTCTAATTTGAGATTACTACAATGAGCGAA  GATCCCTAGGGATTCCTTGATCAGGAAATCTTCCAG | qRT-PCR  qRT-PCR  qRT-PCR  qRT-PCR  qRT-PCR  qRT-PCR  qRT-PCR  qRT-PCR  qRT-PCR  qRT-PCR  qRT-PCR  qRT-PCR  qRT-PCR  qRT-PCR  qRT-PCR  qRT-PCR  qRT-PCR  qRT-PCR  qRT-PCR  qRT-PCR  qRT-PCR  qRT-PCR  qRT-PCR  qRT-PCR  qRT-PCR  qRT-PCR  qRT-PCR  qRT-PCR  qRT-PCR  qRT-PCR  qRT-PCR  qRT-PCR  qRT-PCR  qPCR  qPCR  qPCR  qPCR  qPCR  qPCR  qPCR  qPCR  pASK-Hns-3FLAG  pASK-Hns-3FLAG |
